# 1416. Walk a Mile in Someone Else’s Shoes: Navigating the Complexity of Daily Environmental Cleaning – A Quality Improvement Pilot Project

**DOI:** 10.1093/ofid/ofad500.1253

**Published:** 2023-11-27

**Authors:** Linda McKinley, Julie Keating, Sydney Hoel, Vishala Parmasad, Nasia Safdar

**Affiliations:** Wm. S. Middleton Memorial VA Hospital, Madison, Wisconsin; Madison VA Hospital, Madison, Wisconsin; Madison VA Hospital, Madison, Wisconsin; University of Wisconsin - Madison, Madison, Wisconsin; University of Wisconsin School of Medicine and Public Health, Madison, Wisconsin

## Abstract

**Background:**

A major tenet of environmental cleaning is to focus on high-touch surfaces (HTS). HTS in the patient’s proximity have high rates of contamination, yet cleaning compliance of HTS remains low particularly when the patient is present. This quality improvement (QI) project aimed to use stakeholder input to modify a HTS cleaning checklist.

**Methods:**

This pilot project used a Plan-Do-Check-Act cycle to modify and evaluate a daily cleaning checklist across acute and long-term care settings at one Midwestern VA Hospital (**Figure 1**).

PDCA

**Figure:**
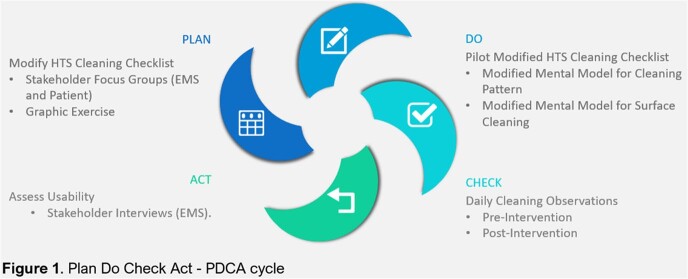

**Results:**

Two pre-intervention focus groups (patients, N=8 and EMS, N=5) were conducted to understand stakeholder perspectives and barriers to daily cleaning to inform modifications to the cleaning checklist. Participants viewed the graphic and discussed patients’ and EMS’s possible notions upon EMS entering the patient room. The groups were then asked how these insights could be addressed during the cleaning process. Recurrent themes of stakeholder’s perceived barriers to cleaning are depicted in **Figure 2**. Integrating the stakeholder suggestions to overcome barriers, and the literature suggesting higher touch and contamination of surfaces within proximity to the patient, the QI team and EMS designed a paradigm shift for the checklist including modifying mental models for: a. cleaning patterns from clean-to-dirty to dirty-to-clean (**Figure 3**) and b. extent of surface cleaning (**Figure 4**).

Pre- and post-intervention observations of daily cleaning compliance of 10 HTS showed a significant improvement of 38% (pre, N=7) to 72% (post, N=10) (p = 0.011).

Two EMS interviews were conducted post-intervention to evaluate checklist usability. Emergent themes were discomfort with cleaning any surface near the patient while the patient was in the bed and with the use of the modified cleaning pattern due to discordance with previous training and practices

Graphic Exercise

**Figure:**
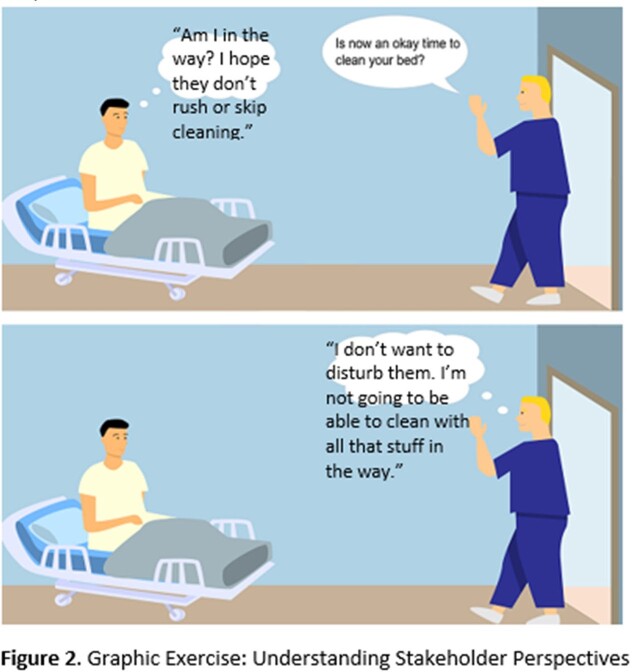

Cleaning Pattern Mental Model

**Figure:**
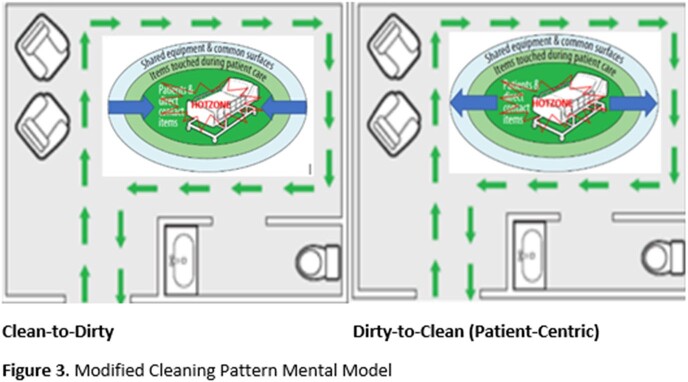

Modified Checklist

**Figure:**
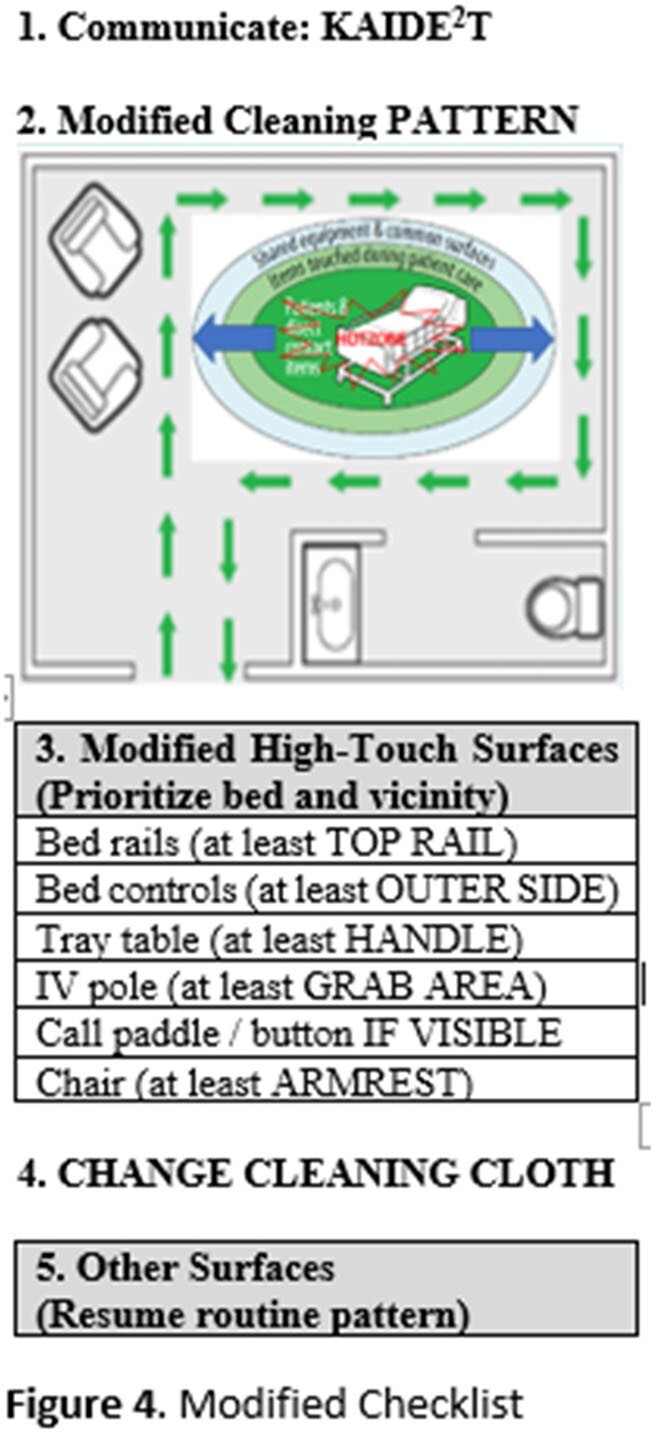

Modified Checklist

**Conclusion:**

Current checklists do not address contextual factors of navigating the environment surrounding patients in bed. HTS cleaning rates may be improved by modifying checklists based on input from local stakeholders. Future research is needed to evaluate modified cleaning processes to facilitate daily cleaning of occupied patient rooms.

**Disclosures:**

**All Authors**: No reported disclosures

